# Parental Acceptance and Control as Predictors of Different Forms of Aggression Among Turkish Emerging Adults

**DOI:** 10.3390/bs16060870

**Published:** 2026-05-31

**Authors:** Ertuğrul Şahin, Abdullah Manap, Nursel Topkaya

**Affiliations:** 1 Department of Guidance and Psychological Counseling, Faculty of Education, Amasya University, 05100 Amasya, Türkiye; 2 Department of Psychology, Faculty of Arts and Sciences, Batman University, 72100 Batman, Türkiye; abdullah.manap@batman.edu.tr; 3 Department of Guidance and Psychological Counseling, Faculty of Education, Çanakkale Onsekiz Mart University, 17100 Çanakkale Merkez, Türkiye; nursel.topkaya@comu.edu.tr

**Keywords:** parental acceptance, parental control, physical aggression, verbal aggression, anger, hostility, general aggression, emerging adults, Türkiye

## Abstract

Previous studies have predominantly examined parental acceptance, parental control, and aggression separately, often focusing on one parenting dimension or one parental figure at a time. This study investigated the associations between maternal, paternal, and parental acceptance and control and five distinct forms of aggression (physical aggression, verbal aggression, anger, hostility, and general aggression) among Turkish emerging adults. A convenience sample of 487 emerging adult university students (381 females, 106 males) completed the Personal Information Form, the Parenting Styles Questionnaire, and the Buss-Perry Aggression Questionnaire. Data were analyzed using descriptive statistics, Pearson product-moment correlation analyses, and hierarchical regression analyses. Results indicated that maternal acceptance and parental acceptance were negatively associated with physical aggression, verbal aggression, hostility, and general aggression, whereas paternal acceptance was negatively associated with hostility and general aggression. Maternal control, paternal control, and overall parental control consistently emerged as significant positive predictors of physical aggression, verbal aggression, anger, hostility, and general aggression. These findings suggest that parental control is associated with higher levels of various forms of aggression among Turkish emerging adults, whereas parental acceptance is associated with lower levels of aggression, with differential effects of maternal and paternal parenting. Overall, our findings indicate that parenting influences on aggression during emerging adulthood are multifaceted, varying by parenting dimension, parental figure, and form of aggression. Interventions for emerging adults should prioritize reducing excessive parental control as a universal target regardless of parental source, while fostering parental acceptance (particularly maternal acceptance) may be associated with additional protective effects for most but not all forms of aggression.

## 1. Introduction

Aggression is broadly defined as behavior aimed at harming others physically or psychologically (Gavin & Porter, 2015). The multifaceted nature of aggression has led to diverse explanations across disciplines. While a general definition exists, many researchers argue for a multidimensional approach, recognizing different types of aggression that require distinct definitions (Allen & Anderson, 2017). The existing literature presents various classifications of aggression, reflecting its complex nature. This study adopts the framework proposed by Buss and Perry (1992), which delineates four forms of aggression: physical, verbal, anger, and hostility. Physical aggression involves harmful actions, such as pushing, pulling, or hitting, directed at another person or an object. Verbal aggression, in contrast, manifests as harmful speech directed at others. Hostility reflects enduring negative attitudes, cynicism, and mistrust toward others. It represents a cognitive predisposition that may lead to aggression. Anger is an emotional state involving physiological arousal and feelings of displeasure. It typically arises in response to perceived injustices, provocations, frustrations, or threats (Allen & Anderson, 2017; Buss & Perry, 1992; Gavin & Porter, 2015; Krahé, 2020). In addition to these specific forms of aggression, general aggression is used as a composite measure representing an individual’s overall tendency to act aggressively across cognitive, emotional, and behavioral domains.

While adolescence is traditionally considered a critical period for aggressive behavior (Santrock, 2018; Steinberg, 2022), emerging adulthood (ages 18 to 29) is an equally important yet understudied stage of development. It is characterized by identity exploration, instability, and the transition from adolescent dependence to adult responsibility (Arnett, 2000; Arnett et al., 2014). Given the ongoing brain maturation and establishment of long-term behavioral patterns during this period, understanding aggressive behavior is essential for designing effective interventions (Arnett, 2000; Arnett et al., 2014). Despite its importance, research examining the relationship between parenting practices and multidimensional aggression in emerging adults remains limited.

Aggressive behavior is a learned trait, often established early in life, with family dynamics playing a pivotal role in its development (Krahé, 2020). A harmonious family environment fosters positive developmental outcomes in children, whereas adverse family experiences and persistent negative interactions can have lasting detrimental effects for both parents and children (Pinquart, 2017; Pinquart & Kauser, 2018; Rohner & Khaleque, 2010; Rothenberg et al., 2020). Parental behaviors play a critical role in shaping children’s personality development, self-concept, and worldview from early childhood (Khaleque, 2021; Smetana, 2017). Supportive parenting, characterized by warmth and encouragement, is associated with positive developmental outcomes, while negative parenting practices, such as neglect or harsh discipline, are linked to increased emotional and behavioral issues (Pinquart, 2017; Pinquart & Kauser, 2018; Rothenberg et al., 2020).

This study is based on two theoretical frameworks. According to Parental Acceptance-Rejection Theory (PARTheory; Rohner et al., 2005; Rohner & Khaleque, 2010), perceived parental acceptance fosters emotional security, a positive self-concept, and adaptive behavioral outcomes. In contrast, parental rejection has been associated with hostility, aggression, and psychological maladjustment. PARTheory suggests that these effects are universal across cultures but may manifest differently depending on the cultural context. Self-Determination Theory (SDT; Ryan & Deci, 2018) posits that individuals have three core psychological needs, namely relatedness, autonomy, and competence. According to SDT, such needs are unlikely to be fulfilled when parents engage in high levels of psychological and behavioral control, which has been associated with poor self-regulation, manifesting as resentment, frustration, and aggression. On the other hand, warmth and acceptance by parents have been associated with the fulfillment of the need for relatedness and competence, thereby enhancing adaptive regulation of emotions and behavior. These two dimensions of control and acceptance are thought to work independently and additively to affect child outcomes (Baumrind, 2013; Rohner et al., 2005).

Research has consistently shown that parenting practices significantly impact the development of aggression (Baumrind, 2013; Masud et al., 2019). Parental acceptance and control are particularly important constructs in this regard. Within the framework of parenting dimensions, parental acceptance refers to the warmth, affection, care, comfort, concern, nurturance, support, or love that parents express toward their children (Rohner et al., 2005). It is characterized by behaviors such as showing physical and verbal affection, offering praise and encouragement, actively listening to the child’s needs, and providing emotional support. In contrast, parental control encompasses the rules and restrictions that parents impose on their children, as well as the degree to which they monitor their children’s activities. It can be further divided into behavioral control, which involves setting rules and monitoring behavior, and psychological control, which includes manipulation of the child’s emotions and thoughts (Barber, 1996).

Studies of parenting practices conducted across different cultural contexts have shown that parental acceptance is generally associated with lower levels of aggression (Muris et al., 2004; Özdemir et al., 2017; Pinquart, 2017; Rothenberg et al., 2020), while psychological control, harsh discipline, and emotional rejection are generally associated with higher levels of aggression (Batool & Bond, 2015; Chen et al., 2020; Meesters et al., 1995; Nicholas & Bieber, 1996; Pinquart, 2017). Categorical (e.g., authoritative, authoritarian, permissive, and neglectful) and dimensional (e.g., parental acceptance and control) parenting practice approaches also generally show consistent patterns linking parenting practices with various forms of aggression, including physical, verbal, anger, relational, hostility, and general aggression, across diverse samples (e.g., Batool & Bond, 2015; Llorca et al., 2017; Masud et al., 2019; Muris et al., 2004). However, questions remain unanswered about how these associations may differ across different developmental periods and across different parental figures.

A growing body of research also underscores the need to distinguish between maternal and paternal parenting influences on different forms of aggressive behavior across diverse samples. For example, Kawabata and Crick (2016) found gender-specific parental effects on aggressive behavior among Japanese children. Specifically, maternal relational aggression predicted physical aggression in all children, but relational aggression only in boys. However, only paternal, not maternal, conflict predicted peer-directed physical aggression. Ingram et al. (2019) demonstrated that paternal care was associated with anger and verbal aggression in young adult males, whereas maternal care was associated with physical aggression in young adult females. Additionally, maternal overprotection was associated with hostility in young adult males. These findings suggest that the relationship between parenting behaviors and aggressive outcomes varies systematically according to parent and child gender, such that maternal and paternal influences predict different forms of aggressive behavior.

Understanding parenting practices in the Turkish context requires recognizing Türkiye’s unique cultural position as a bridge between Eastern and Western values. Türkiye is a relatively collectivist culture with autonomous-relational characteristics where traditional values coexist with modernization processes (Cindoglu et al., 2008; Kağıtçıbaşı, 2005). Research suggests that moderate parental control is normative in Turkish culture and may be perceived as an expression of care rather than rejection (Erkman & Rohner, 2006). However, parental acceptance and parental control have been independently associated with child outcomes across diverse cultural settings, including collectivistic cultures (Pinquart & Kauser, 2018). Traditional Turkish culture emphasizes distinct parenting roles, with mothers primarily responsible for emotional nurturing and protection and fathers primarily responsible for authority and control (Gürmen & Kılıç, 2022). Nevertheless, rapid social changes, including increased enrollment in higher education and greater exposure to Western cultural influences, are reshaping family dynamics among Turkish emerging adults (Çelik et al., 2025; Gürmen & Kılıç, 2022). Despite the importance of understanding parenting influences in this unique context of cultural transition, research examining parental acceptance, control, and aggression among Turkish emerging adults remains limited. Most studies of correlates of aggression focus on children or adolescents (e.g., Baş & Yurdabakan, 2012; Çevik et al., 2021; Özdemir et al., 2017) and leave a significant gap regarding how parenting dimensions influence aggression in emerging adulthood.

Several important gaps in the existing research make this study necessary. First, while extensive research has examined parenting effects during childhood and adolescence, emerging adulthood represents a distinct developmental period characterized by decreased parental supervision and continued parental influence, which together may interact differently with parenting dimensions (Arnett, 2000; Arnett et al., 2014). Second, previous studies have often examined parental acceptance and control independently, without simultaneously investigating the unique and differential contributions of both dimensions across multiple distinct forms of aggression. Most research has either examined only maternal or paternal parenting or aggregated maternal and paternal effects without distinguishing between them (e.g., Chen et al., 2020; Meesters et al., 1995; Muris et al., 2004; Nicholas & Bieber, 1996; Rothenberg et al., 2020). This is an important limitation because maternal and paternal parenting roles differ theoretically and empirically (Yaffe, 2023), and moderate correlation between maternal and paternal scores does not rule out differential predictive effects on specific outcomes (Ingram et al., 2019; Kawabata & Crick, 2016). Therefore, it is necessary to examine maternal-only, paternal-only, and composite parental models within the same sample to determine whether aggregation masks significant parent-specific relationships with different forms of aggression.

In addition to parenting dimensions, several other sociodemographic factors have been identified in the literature as potential covariates when examining aggression. Sex differences in aggressive behavior have been widely examined among university students using the Buss-Perry Aggression Questionnaire, with results indicating that males score higher than females on physical aggression and total aggression across diverse cultures (Buss & Perry, 1992; Nakano, 2001; Ramirez et al., 2001; Webster et al., 2014). However, the results concerning verbal aggression, hostility, and anger are inconsistent. Studies on verbal aggression and hostility show that male students tend to score higher than females on both measures (Buss & Perry, 1992; Ramirez et al., 2001; Webster et al., 2014), although some studies have found no sex differences in these forms of aggression (Bernstein & Gesn, 1997; Fossati et al., 2003; Harris & Knight-Bohnhoff, 1996). In terms of anger, there appears to be more inconsistency, with findings suggesting either no sex difference (Archer et al., 1995; Buss & Perry, 1992; Webster et al., 2014) or higher anger levels among females (García-León et al., 2002), with one exception where anger was found to be significantly higher among males (Smith & Waterman, 2006). Because sex differences are not consistent across forms of aggression, sex was included as a control variable to account for its potential differential influence on various aggression outcomes.

Age and parental educational attainment are other sociodemographic factors that have been considered significant in the context of aggression. Studies have revealed that physical forms of aggression typically decrease with age (Tremblay et al., 2018). Studies using the Buss-Perry Aggression Questionnaire have also shown that younger age is positively correlated with higher levels of physical aggression, verbal aggression, hostility, and anger in university students (Harris, 1996). Parental education level is also a relevant variable because parents with higher levels of education tend to be more sensitive and supportive of their children’s autonomy (Davis-Kean et al., 2021; Su-Russell & Russell, 2021). Conversely, children from families with less parental education tend to exhibit higher levels of physical aggression, verbal aggression, hostility, and anger (Lone, 2025). Thus, age, mother’s and father’s education levels are also included as control variables to account for their potential differential influence across aggression outcomes.

Understanding these dynamics is especially important in the Turkish cultural context, where traditional values can influence parenting styles differently from those in Western societies (Ataca, 2009; Cindoglu et al., 2008; Gürmen & Kılıç, 2022). Authoritarian control, emphasizing strict discipline and obedience, is more normative in Türkiye (Ataca, 2009; Gürmen & Kılıç, 2022), though the associations between parental control and child outcomes may vary depending on cultural context (Erkman & Rohner, 2006). However, as Turkish society undergoes rapid modernization and emerging adults are increasingly exposed to Western individualistic values emphasizing personal autonomy, traditional parenting practices emphasizing control may be associated with intergenerational tensions (Cindoglu et al., 2008; Gürmen & Kılıç, 2022). Research indicates that higher levels of parental control are independently associated with increased aggression (Pinquart, 2017), as emerging adults may experience frustration and anger when their autonomy is restricted (Baumrind, 2013; Ryan & Deci, 2018). This is particularly relevant for Turkish emerging adults navigating cultural tensions between traditional collectivist family values and emerging individualistic orientations.

Although previous research has documented the fundamental effects of parental acceptance and control on aggression, several gaps in the literature remain. First, most existing studies have examined acceptance and control separately rather than simultaneously. This makes it difficult to determine the unique contribution of each dimension while accounting for the other. Second, previous studies have rarely distinguished among the effects of maternal acceptance and control, paternal acceptance and control, and combined parental acceptance and control on multiple forms of aggression within the same sample. Third, research on parenting and aggression in young adulthood is limited in non-Western cultural contexts, such as Türkiye. This study simultaneously addresses all three of these gaps by examining the unique and independent predictive effects of both parental acceptance and parental control on five distinct forms of aggression while distinguishing among maternal, paternal, and combined parental effects. Therefore, the objective of the present study is to investigate the associations between maternal, paternal, and parental acceptance and control with different forms of aggression (physical aggression, verbal aggression, anger, hostility, and general aggression) among Turkish emerging adults.

Consistent with the conceptualization of acceptance and control as theoretically independent dimensions (Baumrind, 2013; Rohner et al., 2005), the present study examined their unique additive contributions to different forms of aggression. Based on theoretical predictions and a review of the literature, the following hypotheses were formulated. First, maternal, paternal, and combined parental control were hypothesized to be positive predictors of physical aggression, verbal aggression, anger, hostility, and general aggression. Second, maternal, paternal, and combined parental acceptance were hypothesized to be negative predictors of physical aggression, verbal aggression, anger, hostility, and general aggression. Third, it was hypothesized that maternal and paternal parenting would differentially predict distinct forms of aggression. Specifically, it was expected that maternal acceptance would demonstrate broader protective effects against aggression than paternal acceptance in the Turkish cultural context.

## 2. Methods

### 2.1. Research Design

This study employed a cross-sectional research design to examine associations of maternal, paternal, and combined parental acceptance and control with physical aggression, verbal aggression, anger, hostility, and general aggression among Turkish emerging adults while controlling for gender, age, and parental education levels (Adu & Miles, 2024).

### 2.2. Participants

We conducted a priori power analysis to determine the minimum sample size required to detect a small-to-medium effect size (*R*^2^ = 0.08) with a statistical power of 0.95 and a significance level of 0.01, considering the inclusion of six independent variables in the hierarchical regression analyses. The power analysis showed that a minimum of 348 participants was necessary for this study to achieve the desired power (Faul et al., 2009). To enhance the representativeness of the sample and to account for potential attrition and missing data, we aimed to recruit at least 400 emerging adults. The selection of a small-to-medium effect size was informed by previous empirical findings on the relationship between parenting styles and various forms of aggression (e.g., Batool, 2013; Batool & Bond, 2015; Meesters et al., 1995; Nicholas & Bieber, 1996; Pinquart, 2017; Rothenberg et al., 2020).

Inclusion criteria required participants to be emerging adult university students whose parents were both alive and who had not separated before the participants turned 18. We screened 618 university students and excluded 131 for the following reasons: Twenty-nine participants experienced the death of a parent before the age of 18 (5 mothers, 23 fathers, and 1 who lost both parents); 50 had parents who separated or divorced before the age of 18; 23 were outside the target age range (18–29 years); 16 failed to answer attention-check items correctly (for example, “Please answer this question as somewhat uncharacteristic of me”); and 13 were excluded as outliers (5 univariate and 8 multivariate). This resulted in 487 emerging adult university students being included in the final analysis. We recruited participants using convenience sampling (Adu & Miles, 2024). This method involves selecting participants based on their accessibility and proximity to the researchers, rather than through random selection from the entire population of interest. The final sample comprised 381 females (78.2%) and 106 males (21.8%). The ages of emerging adults ranged from 18 to 29 years, with a mean of 21.49 years (*SD* = 2.18). Participants were distributed across university years as follows: preparatory year (*n* = 17, 3.5%), first year (*n* = 60, 12.3%), second year (*n* = 106, 21.8%), third year (*n* = 202, 41.5%), fourth year (*n* = 79, 16.2%), fifth year (*n* = 14, 2.9%), and sixth year (*n* = 9, 1.8%). Completed maternal education (in years) ranged from 0 to 19 (*M* = 7.78, *SD* = 4.13); the most common level was 5 years (equivalent to primary school, *n* = 178, 36.6%). Years of completed paternal education ranged from 0 to 19 (*M* = 9.65, *SD* = 3.92); the most common level was 5 years (equivalent to primary school; *n* = 137, 28.1%).

### 2.3. Measures

Personal Information Form: This form was used to collect sociodemographic information from participants. Participants were asked to provide information regarding their gender, age, year of study, and maternal and paternal education levels.

Buss–Perry Aggression Questionnaire: To assess different forms of aggression, we administered the Buss–Perry Aggression Questionnaire (BPAQ; Buss & Perry, 1992), adapted into Turkish by Demirtaş Madran (2013). The 29-item BPAQ comprises four subscales and can be used to assess either total aggression or specific forms. Participants rate each item on a 5-point Likert scale ranging from extremely uncharacteristic of me (1) to extremely characteristic of me (5). Score ranges were 9–45 for physical aggression, 5–25 for verbal aggression, 7–35 for anger, 8–40 for hostility, and 29–135 for the total score. Higher scores indicate greater levels of aggression. The Turkish version of BPAQ demonstrated a four-factor structure similar to that of the original questionnaire, accounting for 41.4% of the variance among adolescents. Cronbach’s alpha coefficients for the subscales demonstrated marginal to good internal consistency ranging from 0.48 (verbal aggression) to 0.78 (physical aggression) in the Turkish version of BPAQ (Demirtaş Madran, 2013). In this study, Cronbach’s alpha values were 0.85 for the physical aggression subscale, 0.60 for the verbal aggression subscale, 0.80 for the anger subscale, 0.72 for the hostility subscale, and 0.90 for the total scale. The relatively low internal consistency (α = 0.60) of the verbal aggression subscale in this study is comparable to that in prior studies across various cultural contexts (Meesters et al., 1996; Morren & Meesters, 2002; von Collani & Werner, 2005; Zimonyi et al., 2021). A sample item from the physical aggression subscale is “Given enough provocation, I may hit another person”; from the verbal aggression subscale, “My friends say that I’m somewhat argumentative”; from the anger subscale, “I have trouble controlling my temper”; and from the hostility subscale, “I am suspicious of overly friendly strangers”.

Parenting Styles Questionnaire: This study utilized the Parenting Styles Questionnaire developed by Sümer and Güngör (1999) to assess parental attitudes and behaviors. The Parenting Styles Questionnaire was selected for this study due to its cultural relevance and appropriateness. Türkiye, like many other countries, has a unique family structure and parenting norms that may differ from those in Western contexts (Ataca, 2009; Gürmen & Kılıç, 2022). Using a measure validated in the Turkish cultural context can ensure that the questionnaire accurately reflects parenting practices and beliefs. This instrument comprises 22 identical items for mothers and fathers, divided into two dimensions: acceptance, interest, and love; and strict supervision and control. Each dimension contains 11 items. Participants are required to respond to each question by reflecting on their childhood experiences and on their relationship with their parents in general. The questionnaire employs a 5-point Likert-type scale ranging from 1 (not true at all) to 5 (very true), with dimension scores ranging from 11 to 55. Reliability analysis yielded Cronbach’s alpha (α) values of 0.91, 0.86, 0.92, and 0.81 for maternal acceptance, maternal control, paternal acceptance, and paternal control, respectively, in this study.

In addition to individual scores for mothers and fathers, composite parental scores were used in this study for two primary reasons. First, Pearson correlation analyses revealed moderate-to-high positive correlations between maternal and paternal scores in both dimensions (Acceptance: *r* = 0.43, *p* < 0.001; Control: *r* = 0.53, *p* < 0.001), indicating that emerging adults’ assessments of maternal and paternal attitudes were not independent. Second, we conducted a confirmatory factor analysis (CFA) using mean- and variance-adjusted maximum likelihood estimation (MLMV) to determine whether we could validly combine maternal and paternal scores into composite parental scores. The hypothesized two-factor model (parental acceptance and parental control) demonstrated a good fit to the data (χ^2^[1] = 4.29, Comparative Fit Index = 0.99, Tucker–Lewis Index = 0.92, Standardized Root Mean Square Residual = 0.02, and Root Mean Square Error of Approximation = 0.08, *p* > 0.05, 90% CI [0.02, 0.17]). All factor loadings were significant (*p* < 0.001) and ranged from 0.52 to 0.94. These results support our decision to create composite parental scores by averaging maternal and paternal scores for each dimension. Possible scores range from 11 to 55 for both parental acceptance and parental control. Higher scores on the two subscales indicate greater parental acceptance and control, respectively. A sample item from maternal/paternal acceptance is “I have always trusted my mother’s/my father’s love and affection.” A sample item from maternal/paternal control is “My mother/father wanted to control my every behavior strictly”.

### 2.4. Procedure

We obtained ethical approval for this research from the Scientific Research and Publication Ethics Committee at Çanakkale Onsekiz Mart University. Throughout the study, we adhered to strict ethical standards to ensure the protection of all participants. We collected data primarily from university students in the emerging-adult age range at Amasya University and Çanakkale Onsekiz Mart University. With prior permission from course instructors, we approached participants during regular class hours. Additionally, we asked participants to share the survey link with their friends who were emerging-adult university students to increase sample size.

We collected data using Google Forms, a secure web-based survey platform. We distributed the survey link to participants in person via QR codes or direct links shared in classrooms and through digital channels, such as email, messaging applications, and social media platforms. To minimize order effects and response bias, we randomized the order of all items on the data collection instrument. Before accessing the survey, we presented participants with an informed consent page as the first section of the Google Form. This page provided comprehensive information about the study’s purpose, procedures, potential risks, and benefits. We explicitly informed participants of their right to withdraw from the study at any time without repercussions. Participants could proceed to the survey questions only after providing informed consent. We ensured participant anonymity by disabling the collection of any personally identifiable information in the Google Form settings. Participants completed the survey in approximately 20 min.

### 2.5. Statistical Analysis

The statistical analyses were performed using SPSS 27 software. Prior to analysis, data accuracy, missing values, outliers, and statistical assumptions were examined (Hair et al., 2019; Tabachnick & Fidell, 2019). All variables were within expected ranges. We found no missing data for any variable in the dataset. We identified univariate outliers using standardized *z*-scores (|*z*| > 3.29; Tabachnick & Fidell, 2019) and multivariate outliers using Mahalanobis distance, Cook’s distance, and leverage values (Hair et al., 2019; Tabachnick & Fidell, 2019). We excluded cases identified as outliers by two or more multivariate outlier-detection methods. This process resulted in the removal of five univariate and eight multivariate outliers. Following data cleaning and preparation, the final sample for this study comprised 487 emerging adults.

We calculated descriptive statistics, including frequencies, percentages, means, and standard deviations, to describe the sociodemographic characteristics of emerging adults. We computed Pearson product-moment correlation coefficients to examine the strength and direction of linear relationships among sociodemographic variables (gender, age, maternal education level, and paternal education level); parenting-practice dimensions (maternal acceptance, paternal acceptance, parental acceptance, maternal control, paternal control, and parental control); and aggression outcomes (physical aggression, verbal aggression, anger, hostility, and total aggression). We conducted hierarchical regression analyses to examine the incremental predictive power of parenting practice dimensions for aggression-related outcomes. We conducted three separate hierarchical regression models for each aggression outcome (physical aggression, verbal aggression, anger, hostility, and total aggression). Step 1 of each model included the same sociodemographic variables: gender, age, maternal and paternal education levels. Step 2 varied across the three models: the first model included maternal acceptance and control, the second included paternal acceptance and control, and the third included combined parental acceptance and control. Thus, each model tested the incremental predictive contribution of a distinct set of parenting variables beyond the sociodemographic variables. Each hierarchical regression model tested a different set of predictors, thereby preventing multicollinearity among the maternal, paternal, and composite scores. This design allowed us to examine the unique predictive contribution of each parental figure, test the predictive utility of the composite parental score, and address the lack of research comparing maternal and paternal effects within the same study. Preliminary analyses were also conducted to examine the assumptions of normality, linearity, homoscedasticity, and multicollinearity, which were found to be met (Hair et al., 2019; Tabachnick & Fidell, 2019). The significance level was set at *p* ˂ 0.05 for all inferential analyses.

## 3. Results

### 3.1. Pearson Product-Moment Correlation Analyses

Table 1 presents Pearson product-moment correlation coefficients between sociodemographic variables (gender, age, mother’s and father’s education levels) and aggression outcomes (physical aggression, verbal aggression, anger, hostility, and total aggression) and between parenting dimensions (maternal, paternal, and parental acceptance; maternal, paternal, and parental control) and aggression outcomes among Turkish emerging adults.

As presented in Table 1, physical aggression was weakly positively correlated with gender (*r* = 0.28), maternal control (*r* = 0.18), paternal control (*r* = 0.20), and parental control (*r* = 0.22) and weakly negatively correlated with age (*r* = −0.18), maternal acceptance (*r* = −0.18), and parental acceptance (*r* = −0.15). Verbal aggression was weakly positively correlated with gender (*r* = 0.14), maternal education level (*r* = 0.09), maternal control *(r* = 0.16), paternal control (*r* = 0.17), and parental control (*r* = 0.19) and weakly negatively correlated with age (*r* = −0.10) and maternal acceptance (*r* = −0.11). Anger demonstrated weak positive correlations with maternal control (*r* = 0.15), paternal control (*r* = 0.18), and parental control (*r* = 0.19) and weak negative correlations with maternal acceptance (*r* = −0.18) and parental acceptance (*r* = −0.15). Hostility showed weak positive correlations with maternal control (*r* = 0.28), moderate positive correlations with paternal control (*r* = 0.34) and parental control (*r* = 0.35), and weak negative correlations with maternal acceptance (*r* = −0.24), paternal acceptance (*r* = −0.24), and parental acceptance (*r* = −0.28). Total aggression was weakly positively correlated with gender (*r* = 0.11), maternal control (*r* = 0.25), and paternal control (*r* = 0.28); moderately positively correlated with parental control (*r =* 0.30); and weakly negatively correlated with age (*r* = −0.14), maternal acceptance (*r* = −0.23), paternal acceptance (*r* = −0.14), and parental acceptance (*r* = −0.22).

### 3.2. Hierarchical Multiple Regression Analyses

We conducted a series of hierarchical multiple regression analyses to investigate the relationship between parenting dimensions (maternal acceptance and control, paternal acceptance and control, and combined parental acceptance and control) and five aggression outcomes (physical, verbal, anger, hostility, and total aggression) among Turkish emerging adults. We tested three separate models for each outcome. We present the results of hierarchical multiple regression analyses in Table 2, Table 3, Table 4, Table 5 and Table 6. We entered gender, age, and mother’s education level, and father’s education in Step 1 of all models. These sociodemographic variables accounted for varying amounts of variance across the five outcomes. We found that sociodemographic variables explained 11% of the variance in physical aggression (*F*[4, 482] = 15.04, *p* < 0.001), 3% of the variance in verbal aggression (*F*[4, 482] = 4.41, *p* = 0.002), 1% of the variance in anger (*F*[4, 482] = 1.32, *p* > 0.05), 2% of the variance in hostility (*F*[4, 482] = 1.84, *p* > 0.05), and 3% of the variance in total aggression (*F*[4, 482] = 3.92, *p* = 0.004).

We found that gender and age consistently predicted physical, verbal, and total aggression across all three models. Males (β = 0.11 to 0.28) and younger individuals (β = 0.13 to 0.17) showed higher levels of physical, verbal, and total aggression. We found that mother’s education level negatively predicted hostility (β = 0.12), whereas father’s education level was not a significant predictor in any model.

When parenting variables were added in Step 2, all three models demonstrated statistically significant improvements in explanatory power for physical aggression. The maternal model explained an additional 5% of the variance (Δ*R*^2^ = 0.05, Δ*F*[2, 480] = 13.11, *p* < 0.001); the paternal model explained an additional 4% of the variance (Δ*R*^2^ = 0.04, Δ*F*[2, 480] = 11.75, *p* < 0.001); and the parental model explained an additional 5% of the variance (Δ*R*^2^ = 0.05, Δ*F*[2, 480] = 15.67, *p* < 0.001) in physical aggression scores. The final physical aggression models accounted for 16% (*F*[6, 480] = 14.90, *p* < 0.001, *R*^2^ = 0.16), 15% (*F*[6, 480] = 14.38, *p* < 0.001, *R*^2^ = 0.15), and 17% (*F*[6, 480] = 15.82, *p* < 0.001, *R*^2^ = 0.17) of the total variance for the maternal, paternal, and parental models, respectively. All final parental models had medium effect sizes. In the maternal model, maternal acceptance negatively predicted physical aggression (β = −0.12), whereas maternal control positively predicted physical aggression (β = 0.14). In the paternal model, only paternal control emerged as a positive predictor (β = 0.18), whereas paternal acceptance was not significant (β = −0.06). In the parental model, we found that both parental acceptance (β = −0.10) and parental control (β = 0.18) significantly predicted physical aggression.

Parenting variables were added to each model in Step 2, and all three demonstrated statistically significant improvements in the explanatory power for verbal aggression. The maternal model explained an additional 3% of the variance (Δ*R*^2^ = 0.03, Δ*F*[2, 480] = 6.89, *p* < 0.001); the paternal model explained an additional 3% of the variance (Δ*R*^2^ = 0.03, Δ*F*[2, 480] = 7.19, *p* < 0.001); and the parental model explained an additional 4% of the variance (Δ*R*^2^ = 0.04, Δ*F*[2, 480] = 8.94, *p* < 0.001). We found that the final models accounted for 6% (*F*[6, 480] = 5.31, *p* < 0.001, *R*^2^ = 0.06), 6% (*F*[6, 480] = 5.41, *p* < 0.001, *R*^2^ = 0.06), and 7% (*F*[6, 480] = 6.02, *p* < 0.001, *R*^2^ = 0.07) of the total variance in the maternal, paternal, and parental models, respectively. All final parental models had small effect sizes. In the maternal model, we found that only maternal control was a positive predictor of verbal aggression (β = 0.14), whereas maternal acceptance was not a significant predictor (β = −0.05). In the paternal model, only paternal control was a positive predictor (β = 0.17), whereas paternal acceptance was not significant (β = −0.01). In the parental model, we found parental control (β = 0.18) to be the only significant predictor of verbal aggression, whereas parental acceptance was not significant (β = −0.02).

When we added parenting variables in Step 2, the maternal model explained an additional 4% of the variance (Δ*R*^2^ = 0.04, Δ*F*[2, 480] = 10.14, *p* < 0.001); the paternal model explained an additional 4% of the variance (Δ*R*^2^ = 0.04, Δ*F*[2, 480] = 9.34, *p* < 0.001); and the parental model explained an additional 5% of the variance (Δ*R*^2^ = 0.05, Δ*F*[2, 480] = 11.43, *p* < 0.001) in anger scores. The final anger models accounted for 5% (*F*[6, 480] = 4.29, *p* < 0.001, *R*^2^ = 0.05), 5% (*F*[6, 480] = 4.02, *p* < 0.001, *R*^2^ = 0.05), and 6% (*F*[6, 480] = 4.72, *p* < 0.001, *R*^2^ = 0.06) of the total variance in the maternal, paternal, and parental models, respectively. All final parental models had small effect sizes. In the maternal model, we found that maternal acceptance negatively predicted anger (β = −0.14), whereas maternal control positively predicted anger (β = 0.10). In the paternal model, we found that paternal control was the only positive predictor (β = 0.18), while paternal acceptance was not significant (β = −0.04). In the parental model, we found that both parental acceptance (β = −0.09) and parental control (β = 0.16) significantly predicted anger.

When we added parenting variables in Step 2, the maternal model explained an additional 9% of variance (Δ*R*^2^ = 0.09, Δ*F*[2, 480] = 26.68, *p* < 0.001); the paternal model explained an additional 14% of variance (Δ*R*^2^ = 0.14, Δ*F*[2, 480] = 40.08, *p* < 0.001); and the parental model explained an additional 15% of variance (Δ*R*^2^ = 0.15, Δ*F*[2, 480] = 43.73, *p* < 0.001) in hostility scores. The final hostility models accounted for 11% (*F*[6, 480] = 10.25, *p* < 0.001, *R*^2^ = 0.11), 16% (*F*[6, 480] = 14.78, *p* < 0.001, *R*^2^ = 0.16), and 17% (*F*[6, 480] = 16.02, *p* < 0.001, *R*^2^ = 0.17) of the total variance for the maternal, paternal, and parental models, respectively. All final parental models had medium effect sizes. In the maternal model, we found that maternal acceptance negatively predicted hostility (β = −0.15), while maternal control positively predicted hostility (β = 0.23). Similarly, we found that paternal acceptance negatively predicted hostility (β = −0.17), while paternal control positively predicted hostility (β = 0.30). The parental model yielded consistent results: parental acceptance negatively predicted hostility (β = −0.18), whereas parental control positively predicted hostility (β = 0.29).

When we added parenting variables in Step 2, the maternal model explained an additional 8% of the variance (Δ*R*^2^ = 0.08, Δ*F*[2, 480] = 21.81, *p* < 0.001); the paternal model explained an additional 9% of the variance (Δ*R*^2^ = 0.09, Δ*F*[2, 480] = 23.94, *p* < 0.001); and the parental model explained an additional 11% of the variance (Δ*R*^2^ = 0.11, Δ*F*[2, 480] = 29.32, *p* < 0.001) for total aggression scores. The final total aggression models accounted for 11% (*F*[6, 480] = 10.11, *p* < 0.001, *R*^2^ = 0.11), 12% (*F*[6, 480] = 10.84, *p* < 0.001, *R*^2^ = 0.12), and 14% (*F*[6, 480] = 12.69, *p* < 0.001, *R*^2^ = 0.14) of the total variance in the maternal, paternal, and parental models, respectively. These final parental models demonstrated small-to-medium effect sizes. In the maternal model, we found that maternal acceptance negatively predicted total aggression (β = −0.16), while maternal control positively predicted total aggression (β = 0.19). Similarly, we found that paternal acceptance negatively predicted total aggression (β = −0.10), while paternal control positively predicted total aggression (β = 0.26). The parental model yielded consistent results: parental acceptance negatively predicted total aggression (β = −0.13), whereas parental control positively predicted total aggression (β = 0.25).

## 4. Discussion

This study examined the associations between maternal, paternal, and combined parental acceptance and control and various forms of aggression in Turkish emerging adults. Our research revealed several significant patterns in the association between parenting practices and different forms of aggression. First, we found that parental control was a significant positive predictor of physical aggression, verbal aggression, anger, hostility, and general aggression. Second, maternal and paternal acceptance were protective factors against some forms of aggression, specifically physical aggression, anger, hostility, and general aggression, with differences found between maternal and paternal acceptance. Third, our results showed that the role of parental acceptance and control in aggression persisted into emerging adulthood, even when controlling for sociodemographic factors. The findings offered partial support for the hypotheses. The first hypothesis was fully supported: maternal, paternal, and combined parental control positively predicted all five forms of aggression. The second hypothesis received partial support: maternal, paternal, and combined parental acceptance negatively predicted some but not all forms of aggression. The third hypothesis was also partially supported, in that maternal acceptance demonstrated broader protective effects against different forms of aggression than paternal acceptance.

The positive associations between maternal, paternal, and combined parental control and all five forms of aggression observed in this study are consistent with substantial empirical evidence indicating that controlling parenting practices are associated with increased aggression across diverse samples and cultural contexts, particularly in research involving children and adolescents. For example, in a sample of Chinese children aged 8–12 years, Chen et al. (2020) reported that parental psychological control was positively associated with physical aggression. Batool and Bond (2015) found that authoritarian parenting practices positively correlated with physical aggression, verbal aggression, general aggression, and anger in a sample of adolescents. Similarly, Çevik et al. (2021) reported that parental monitoring was a significant positive predictor of proactive aggression among Turkish adolescents. In a sample of undergraduate students, Nicholas and Bieber (1996) reported a significant positive correlation between emotionally abusive behaviors by both parents and hostility levels. Likewise, Meesters et al. (1995) found that university students with high hostility levels perceived greater parental rejection and overprotection compared to their low-hostility counterparts. Our findings also indicate that both maternal and paternal control contribute significantly to aggressive outcomes, with paternal control demonstrating particularly strong associations with hostility and general aggression. This pattern suggests that controlling behaviors from either parent can undermine emerging adults’ sense of autonomy during a developmental period characterized by identity exploration and increasing independence (Arnett, 2000).

In line with SDT predictions (Ryan & Deci, 2018), the significant positive associations between parental control and different forms of aggression indicate that such parenting practices may be associated with adverse impacts on emerging adults’ basic psychological needs, which may be linked to aggression. Physical and verbal aggression may be associated with frustration when emerging adults perceive their autonomy as threatened (Ryan & Deci, 2018). Anger may be associated with the emotional experience of parental intrusion and control. Hostility may be related to the resentment emerging adults feel toward controlling parents. Similarly, consistent with PARTheory (Rohner et al., 2005), high parental control and low parental warmth have each been independently associated with emerging adults feeling unloved and unsupported. These feelings may be associated with physical aggression, verbal aggression as an expression of frustration, anger as a response to perceived parental intrusion, hostility as a sustained negative orientation toward others, and general aggression as a broader tendency to respond aggressively across situations.

The findings that maternal, paternal, and combined parental control predicted increased aggression are particularly noteworthy within the Turkish cultural context. While moderate parental control is normative in Turkish culture and may be perceived as an expression of care rather than rejection (Erkman & Rohner, 2006), the present findings indicate that higher levels of parental control are independently and positively associated with all forms of aggression examined among Turkish emerging adults. This pattern was consistent across maternal, paternal, and combined parental models, suggesting that the association between control and aggression operates independently of levels of parental acceptance. Moreover, both maternal and paternal control independently predicted aggression, highlighting the complementary yet distinct roles that Turkish mothers and fathers play in child-rearing practices. In traditional Turkish family structures, mothers typically exercise psychological control through emotional manipulation and guilt induction, while fathers tend to exert behavioral control through rule enforcement and authority (Ataca, 2009; Cindoglu et al., 2008; Gürmen & Kılıç, 2022). The particularly strong associations found between paternal control and aggression may reflect the authoritative position fathers hold in Turkish families, where paternal authority is culturally emphasized. This finding aligns with recent research indicating that Turkish emerging adults experience tensions between traditional collectivist values emphasizing family authority and increasingly individualistic values promoting personal autonomy (Akyil et al., 2016; Gürmen & Kılıç, 2022). When emerging adults perceive high parental control that restricts their autonomy during this critical developmental period, frustration and aggressive behavior may be more likely, which may be associated with efforts to assert independence (Abaied & Emond, 2013). Our findings extend previous research by demonstrating that these associations persist into emerging adulthood, suggesting that parental control remains associated with aggressive tendencies even as young adults gain greater independence from their families.

Our findings indicate that parental acceptance acts as a protective factor against aggression, with differential effects for maternal and paternal acceptance. Parental and maternal acceptance significantly predicted lower levels of physical aggression, anger, hostility, and general aggression, whereas paternal acceptance predicted only lower levels of hostility and general aggression. These findings are consistent with previous studies demonstrating that low parental acceptance is positively correlated with various forms of aggressive behavior across diverse samples and cultures, primarily among children and adolescents. In a comprehensive meta-analysis, Pinquart (2017) found that parental warmth and autonomy-granting were negatively associated with externalizing behaviors in children and adolescents. Additionally, Rothenberg et al. (2020) reported that parental warmth was associated with fewer externalizing problems and acted as a protective factor in most countries. Batool and Bond (2015) found that authoritative parenting practices were negatively correlated with physical aggression, verbal aggression, hostility, and overall aggression in adolescents. Similarly, Muris et al. (2004) found that maternal and paternal emotional warmth negatively correlated with physical aggression, verbal aggression, anger, hostility, and overall aggression. Özdemir et al. (2017) also reported that maternal closeness was negatively correlated with physical aggression among Turkish adolescents.

Consistent with PARTheory predictions (Rohner et al., 2005; Rohner & Khaleque, 2010), parental acceptance was associated with lower levels of different forms of aggression. According to PARTheory, perceived parental acceptance is associated with a positive self-concept and emotional security (Rohner et al., 2005), which may be linked to more adaptive emotional regulation and a lower likelihood of aggressive responding. More specifically, parental acceptance has been linked to lower physical aggression, which may be associated with more constructive conflict resolution and greater impulse regulation. The association between perceived parental acceptance and lower levels of anger may reflect the emotional security it provides, which in turn may be associated with less intense emotional reactivity when facing frustrations. Similarly, parental acceptance has been associated with lower hostility, potentially through its links with greater interpersonal trust and more positive attributions about others’ intentions and with lower general aggression, potentially through its associations with more prosocial coping tendencies. This pattern appears particularly salient in the Turkish cultural context, where family relationships remain strong even during emerging adulthood.

The broader protective effects of maternal acceptance, compared with paternal acceptance, merit further exploration. This differential pattern may reflect gender-specific parenting roles in Turkish culture, in which fathers traditionally take on authoritative and disciplinary roles, while mothers primarily provide emotional support (Ataca, 2009; Cindoglu et al., 2008; Gürmen & Kılıç, 2022). Previous research has shown that maternal and paternal parenting influences operate through different pathways and exert differential effects on aggressive outcomes (Murray et al., 2014). Our findings suggest that maternal acceptance is particularly important for preventing various forms of aggression, whereas paternal acceptance provides more specific protection against hostility and general aggression. However, the significant associations between paternal control and aggression across all outcomes indicate that paternal parenting practices remain influential during emerging adulthood, particularly with respect to the control dimension rather than the acceptance dimension.

We also found that the association between parental acceptance and verbal aggression was weaker than the associations between parental acceptance and other forms of aggressive behavior. When entered alongside the control variables, none of the three predictor variables (maternal acceptance, paternal acceptance, and parental acceptance) significantly predicted verbal aggression. However, all three control variables were significant positive predictors. These findings suggest that verbal aggression may develop through different mechanisms than other forms of aggression. These findings differ from those of studies conducted with children and adolescents, in which parental acceptance was found to be negatively associated with verbal aggression (Batool, 2013; Batool & Bond, 2015; Muris et al., 2004). While parental acceptance appears to reduce aggression by fostering emotional security and impulse control, these protective factors may be less relevant for verbal aggression, which is primarily a learned communication style acquired through direct modeling and imitation of parental behavior (Cui et al., 2010). The warmth and emotional support provided by accepting parents may not be sufficient to prevent verbal aggression if those parents themselves use verbally aggressive communication. Our findings suggest that parental modeling of controlling behaviors is a more salient predictor of verbally aggressive behavior than parental acceptance among emerging adults. Psychological control has been found to include verbally aggressive behaviors such as criticism, guilt induction, and love withdrawal (Yu et al., 2015). Emerging adults exposed to these controlling and verbally aggressive communication patterns may adopt similar patterns in their own relationships.

The current study provided further insights into the functions of the sociodemographic control variables used in regression analysis. Consistent with previous literature, male emerging adults had higher scores for physical aggression, verbal aggression, and total aggression than female emerging adults (Buss & Perry, 1992; Ramirez et al., 2001; Webster et al., 2014). In line with other findings regarding inconsistent sex differences in anger and hostility among college student samples, no significant differences were found between males and females regarding anger and hostility (Bernstein & Gesn, 1997; Fossati et al., 2003; Harris & Knight-Bohnhoff, 1996). These findings suggest that sex differences may be less pronounced in these forms of aggression than in behaviorally expressed forms of aggression. Age was also a significant negative predictor of physical aggression, verbal aggression, and total aggression, consistent with previous studies indicating that overt forms of aggression tend to decrease as individuals mature and internalize social norms (Harris, 1996; Tremblay et al., 2018). However, age was not a significant predictor of anger or hostility. This may reflect that these forms of aggression are more dispositionally stable and more cognitively entrenched than behaviorally expressed forms. Regarding parental education, mother’s education level was found to be a significant negative predictor of hostility. This may reflect the role that more educated mothers play in positively influencing their children’s perceptions of others and reducing the cynicism and mistrust characteristic of hostility (Lansford et al., 2010; Lone, 2025). In contrast, fathers’ education level did not significantly predict any form of aggression. The absence of significant effects for mother’s education on other aggression outcomes and for father’s education across all outcomes may partly reflect the relatively low and narrow range of parental education in our sample, in which the most common level was five years of completed schooling for both parents.

### 4.1. Practical Implications

The findings of our study have several important practical implications for parents, clinicians, and educators working with emerging adults and their families. Because parenting is associated with aggressive behaviors that persist into emerging adulthood, interventions focusing on parent–child relationships are relevant and effective during this developmental stage. First, parental control was a universal predictor of all five aggression outcomes, and the combined parenting models generally accounted for the most variance. Therefore, interventions should focus on reducing parental control through family-based programs that involve both parents. These interventions could help parents recognize patterns of control, such as psychological control (e.g., guilt induction, love withdrawal, and invalidation of feelings) and excessive behavioral control. They could also help parents develop new, more autonomous, yet nurturing strategies for emerging adults. In the Turkish cultural context, where collectivist values coexist with individualistic tendencies among emerging adults, these interventions need to be culturally sensitive. They should help families find a balance between maintaining the family connectedness valued in collectivist societies and the autonomy characteristic of emerging adulthood. Given that maternal acceptance demonstrated a stronger protective effect while paternal control showed stronger associations with multiple forms of aggression, interventions may also require parent-specific tailoring. Specifically, programs could support mothers in maintaining warm, accepting relationships while fostering independence and help fathers reduce controlling behaviors while remaining positively engaged in their emerging adult children’s lives.

Second, different forms of aggression require different intervention strategies. Hostility was found to have the strongest associations with parenting practices and the greatest explained variance among all forms of aggression examined in our study. These findings suggest that hostile individuals may benefit most from interventions that directly address family dynamics. Specifically, interventions for reducing hostility should prioritize improving family relationships and may benefit from therapeutic approaches that address emotional dysregulation and maladaptive cognitive patterns underlying hostile responses such as acceptance and commitment therapy or mindfulness-based interventions (Byrne & Cullen, 2024; Tao et al., 2021). Conversely, verbal aggression was associated with parental control rather than parental acceptance. These findings suggest that reducing verbal aggression may require interventions focusing on peer influences, communication skills training, and social norms rather than primarily targeting parent–child relationships.

Third, university counseling centers and mental health clinics should incorporate the evaluation of family relationships into their assessments and treatment plans for aggression-related concerns. Mental health professionals should systematically assess parental acceptance and control to identify emerging adults at risk for aggressive behaviors and to provide appropriate psychological services. Mental health professionals may also benefit from helping emerging adults process feelings of parental rejection or control to reduce various forms of aggression; improve emotional regulation to decrease physical aggression and anger; address hostile attribution biases to reduce hostile behaviors; and develop healthier interpersonal and relational patterns to lessen verbal aggression, all within individual therapy sessions. Universities might offer preventive interventions through orientation sessions and workshops that address common challenges in relationships between parents and emerging adults, offer advice on how to set appropriate boundaries while remaining connected, and provide educational materials for parents of university students about this stage of development. Our findings indicate that early parenting practices are associated with aggressive behaviors in emerging adulthood. Accordingly, prevention efforts may benefit from targeting aggressive behaviors before emerging adulthood by implementing early intervention programs that promote positive parenting practices. Specifically, parenting interventions that foster warmth and acceptance while teaching developmentally appropriate and effective behavioral control strategies should be made widely accessible to families.

### 4.2. Limitations

Several limitations should be considered when interpreting our research findings. First, the cross-sectional nature of our study does not allow us to make definitive claims about causality and the direction of effects. Although our theoretical model proposes that parenting practices (acceptance and control) predict different forms of aggression, the reverse direction of effects is also possible. Second, our use of self-report measures is vulnerable to certain biases, such as social desirability, common method variance, and recall bias. The retrospective evaluations of emerging adults on parenting practices may be shaped by their current psychological conditions and may not necessarily correspond to actual parenting practices (McKinney & Kwan, 2018). Similarly, self-reported measures of aggression may be prone to underreporting due to social desirability bias. Furthermore, the low internal consistency of the verbal aggression subscale (α = 0.60) represents a measurement limitation. Although this reliability coefficient is consistent with those reported in other studies (Demirtaş Madran, 2013; Meesters et al., 1996; Morren & Meesters, 2002; von Collani & Werner, 2005; Zimonyi et al., 2021), it may have attenuated the associations between parenting variables and verbal aggression. Thus, findings related to this subscale should be interpreted with caution.

Third, our convenience sample consisted mainly of female university students from two universities in Türkiye, which limits the generalizability of our findings to other populations. The gender distribution in our study (78.2% female) may have limited our ability to detect gender-related patterns in the effects of parenting practices on aggression. University students are a relatively educated and advantaged group of emerging adults and may differ from those who do not attend university in important ways, including socioeconomic status and developmental experiences. Fourth, our study assessed the relationships between parenting practices and aggression at a single point in time during emerging adulthood, possibly overlooking important developmental changes at different points in this stage of life. Our sample primarily consists of individuals in the earliest phase of emerging adulthood. Thus, our findings may not generalize to older emerging adults. Fifth, our parenting variables assessed emerging adults’ general feelings regarding parental acceptance and control rather than situational fluctuations or recent changes in parenting practices. The Parenting Styles Questionnaire asked participants to recall their childhood experiences, which may not accurately represent current parenting practices during emerging adulthood.

Sixth, the operational definition of parental control used in the current study constitutes another limitation. The existing literature typically distinguishes between two types of parental control: behavioral and psychological (Barber, 1996). However, the Parenting Styles Questionnaire used in this study combines these two types of control into one variable. While behavioral and psychological control tend to be highly correlated empirically, the combination of these two types of control into one factor might have prevented the identification of distinct relationships between them and various forms of aggressive behavior. Seventh, our study did not examine mediating processes that could account for the relationship between parenting practices and different forms of aggression. Eighth, our study did not control for other potential influential factors affecting aggressive behavior, such as peer relations, romantic relationships, personality traits, mental health symptoms, substance use, and community violence exposure. The small-to-medium effect sizes found in our regression analyses (explaining approximately 5–17% of variance for different forms of aggression) suggest that parenting variables explain a significant but limited amount of variance in aggression outcomes, which reflects the complex nature of aggressive behavior.

Future studies should aim to address these limitations by employing diverse methodological approaches. First, researchers should conduct longitudinal studies investigating bidirectional relationships between parenting practices and aggression across different age groups to provide substantial evidence regarding developmental processes and mechanisms. In addition to self-report measures, researchers should incorporate multiple informants, such as parents, partners, and peers. To increase external validity, researchers should use more diverse and representative samples that include emerging adults who are not attending higher education, participants from different socioeconomic backgrounds, and equal numbers of both genders. Researchers should also investigate how the relationship between parenting practices and aggression changes across different stages of emerging adulthood. Future researchers should also use measures that enable them to study behavioral and psychological control independently. They should also develop measures specifically designed to assess contemporary parenting styles relevant to emerging adults. Finally, researchers should investigate mediating processes, such as emotion regulation skills, social information processing styles, and attachment representations, to provide a comprehensive understanding of developmental pathways.

## 5. Conclusions

This study examined associations of maternal, paternal, and parental acceptance and control with five different forms of aggression among Turkish emerging adults. We found that parental control functions as a universal risk factor for aggression, showing consistent positive associations across both parental figures and all forms of aggression. Our findings indicate that parental control operates as a transdiagnostic risk factor, significantly predicting physical aggression, verbal aggression, anger, hostility, and general aggression across maternal, paternal, and combined parental models. In contrast, we found that parental acceptance exhibited more selective protective effects, which varied by parental figure and form of aggression. Parental and maternal acceptance protected against physical aggression, anger, hostility, and general aggression; paternal acceptance, however, protected only against hostility and general aggression. Overall, our findings indicate that parenting influences on aggression during emerging adulthood are multifaceted, varying by parenting dimension, parental figure, and form of aggression. We conclude that reducing excessive parental control should be a universal intervention priority for emerging adults, whereas enhancing parental acceptance, particularly maternal acceptance, may provide additional protection against most but not all forms of aggression.

## Figures and Tables

**Table 1:** Pearson Product-Moment Correlations Between Predictor Variables and Aggression Outcomes Among Turkish Emerging Adults.

	Physical Aggression	Verbal Aggression	Anger	Hostility	Total Aggression
	*r*	*r*	*r*	*r*	*r*
1. Gender	0.28 ***	0.14 **	−0.04	−0.05	0.11 *
2. Age	−0.18 ***	−0.10 *	−0.08	−0.05	−0.14 **
3. Mother education level	0.08	0.09 *	0.04	−0.07	0.04
4. Father education level	0.02	0.06	0.00	0.02	0.03
5. Maternal acceptance	−0.18 ***	−0.11 *	−0.18 ***	−0.24 ***	−0.23 ***
6. Maternal control	0.18 ***	0.16 ***	0.15 ***	0.28 ***	0.25 ***
7. Paternal acceptance	−0.08	−0.03	−0.08	−0.24 ***	−0.14 ***
8. Paternal control	0.20 ***	0.17 ***	0.18 ***	0.34 ***	0.28 ***
9. Parental acceptance	−0.15 ***	−0.08	−0.15 ***	−0.28 ***	−0.22 ***
10. Parental control	0.22 ***	0.19 ***	0.19 ***	0.35 ***	0.30 ***

*Note.* Gender: 0 = female, 1 = male. Mother’s and father’s education levels represent completed years of education. *p* < 0.05 *, *p* < 0.01 **, *p* < 0.001 ***.

**Table 2:** Results of Hierarchical Regression Analysis for Physical Aggression Scores.

Variables	*B*	*SE*	β	*t*	*p*
Step 1					
Gender	4.68	0.73	0.27	6.37	0.001 ***
Age	−0.54	0.14	−0.17	−3.91	0.001 ***
Mother education level	0.11	0.09	0.06	1.22	0.223
Father education level	−0.05	0.09	−0.03	−0.57	0.571
Step 2—Maternal Model					
Gender	4.71	0.72	0.28	6.57	0.001 ***
Age	−0.53	0.14	−0.16	−3.89	0.001 ***
Mother education level	0.10	0.09	0.06	1.22	0.221
Father education level	−0.08	0.09	−0.04	−0.86	0.388
Maternal acceptance	−0.11	0.04	−0.12	−2.67	0.008 **
Maternal control	0.12	0.04	0.14	3.04	0.002 **
Step 2—Paternal Model					
Gender	4.62	0.72	0.27	6.41	0.001 ***
Age	−0.55	0.14	−0.17	−4.06	0.001 ***
Mother education level	0.13	0.09	0.07	1.47	0.142
Father education level	−0.06	0.09	−0.03	−0.66	0.509
Paternal acceptance	−0.05	0.03	−0.06	−1.41	0.159
Paternal control	0.17	0.04	0.18	4.21	0.001 ***
Step 2—Parental Model					
Gender	4.67	0.71	0.27	6.55	0.001 ***
Age	−0.55	0.14	−0.17	−4.04	0.001 ***
Mother education level	0.12	0.09	0.07	1.36	0.175
Father education level	−0.07	0.09	−0.04	−0.75	0.455
Parental acceptance	−0.09	0.04	−0.10	−2.14	0.033 *
Parental control	0.19	0.05	0.18	4.12	0.001 ***

*Note.* Gender: 0 = female, 1 = male. Mother’s and father’s education levels represent completed years of education. To preserve space, results from three independent hierarchical regression analyses are presented in a single table. Sociodemographics in Step 1 are identical across all models. *p* < 0.05 *, *p* < 0.01 **, *p* < 0.001 ***.

**Table 3:** Results of Hierarchical Regression Analysis for Verbal Aggression Scores.

Variables	*B*	*SE*	β	*t*	*p*
Step 1					
Gender	1.01	0.34	0.13	2.94	0.003 **
Age	−0.14	0.07	−0.09	−2.10	0.037 *
Mother education level	0.05	0.04	0.07	1.29	0.198
Father education level	0.01	0.04	0.02	0.31	0.755
Step 2—Maternal Model					
Gender	1.01	0.34	0.13	2.98	0.003 **
Age	−0.14	0.06	−0.09	−2.09	0.037 *
Mother education level	0.05	0.04	0.07	1.27	0.203
Father education level	0.01	0.04	0.01	0.18	0.858
Maternal acceptance	−0.02	0.02	−0.05	−1.12	0.263
Maternal control	0.06	0.02	0.14	2.86	0.004 **
Step 2—Paternal Model					
Gender	0.97	0.34	0.13	2.84	0.005 **
Age	−0.14	0.06	−0.10	−2.16	0.031 *
Mother education level	0.06	0.04	0.08	1.50	0.135
Father education level	0.01	0.04	0.01	0.22	0.828
Paternal acceptance	0.00	0.02	−0.01	−0.18	0.860
Paternal control	0.07	0.02	0.17	3.65	0.001 ***
Step 2—Parental Model					
Gender	0.99	0.34	0.13	2.91	0.004 **
Age	−0.14	0.06	−0.10	−2.17	0.030 *
Mother education level	0.06	0.04	0.07	1.40	0.162
Father education level	0.01	0.04	0.01	0.22	0.829
Parental acceptance	−0.01	0.02	−0.02	−0.53	0.597
Parental control	0.08	0.02	0.18	3.75	0.001 ***

*Note.* Gender: 0 = female, 1 = male. Mother’s and father’s education levels represent completed years of education. To preserve space, results from three independent hierarchical regression analyses are presented in a single table. Sociodemographics in Step 1 are identical across all models. *p* < 0.05 *, *p* < 0.01 **, *p* < 0.001 ***.

**Table 4:** Results of Hierarchical Regression Analysis for Anger Scores.

Variables	*B*	*SE*	β	*t*	*p*
Step 1					
Gender	−0.63	0.61	−0.05	−1.05	0.296
Age	−0.20	0.12	−0.08	−1.75	0.080
Mother education level	0.07	0.07	0.06	1.03	0.305
Father education level	−0.04	0.08	−0.03	−0.52	0.601
Step 2—Maternal Model					
Gender	−0.60	0.60	−0.04	−1.01	0.315
Age	−0.19	0.11	−0.07	−1.64	0.102
Mother education level	0.07	0.07	0.06	1.03	0.302
Father education level	−0.06	0.08	−0.04	−0.83	0.404
Maternal acceptance	−0.10	0.03	−0.14	−2.97	0.003 **
Maternal control	0.07	0.03	0.10	2.02	0.044 *
Step 2—Paternal Model					
Gender	−0.69	0.60	−0.05	−1.16	0.247
Age	−0.21	0.11	−0.08	−1.83	0.067
Mother education level	0.09	0.07	0.07	1.25	0.211
Father education level	−0.05	0.08	−0.03	−0.62	0.537
Paternal acceptance	−0.03	0.03	−0.04	−0.93	0.354
Paternal control	0.13	0.03	0.18	3.91	0.001 ***
Step 2—Parental Model					
Gender	−0.63	0.60	−0.05	−1.06	0.290
Age	−0.20	0.11	−0.08	−1.79	0.073
Mother education level	0.08	0.07	0.06	1.13	0.258
Father education level	−0.05	0.07	−0.04	−0.68	0.499
Parental acceptance	−0.07	0.04	−0.09	−2.00	0.046 *
Parental control	0.13	0.04	0.16	3.39	0.001 ***

*Note.* Gender: 0 = female, 1 = male. Mother’s and father’s education levels represent completed years of education. To preserve space, results from three independent hierarchical regression analyses are presented in a single table. Sociodemographics in Step 1 are identical across all models. *p* < 0.05 *, *p* < 0.01 **, *p* < 0.001 ***.

**Table 5:** Results of Hierarchical Regression Analysis for Hostility Scores.

Variables	*B*	*SE*	β	*t*	*p*
Step 1					
Gender	−0.60	0.58	−0.05	−1.05	0.296
Age	−0.13	0.11	−0.05	−1.20	0.232
Mother education level	−0.15	0.07	−0.12	−2.18	0.030 *
Father education level	0.11	0.07	0.08	1.48	0.141
Step 2—Maternal Model					
Gender	−0.58	0.55	−0.05	−1.06	0.288
Age	−0.12	0.10	−0.05	−1.15	0.252
Mother education level	−0.15	0.07	−0.12	−2.34	0.020 *
Father education level	0.08	0.07	0.06	1.19	0.233
Maternal acceptance	−0.10	0.03	−0.15	−3.16	0.002 **
Maternal control	0.15	0.03	0.23	4.91	0.001 ***
Step 2—Paternal Model					
Gender	−0.63	0.54	−0.05	−1.17	0.244
Age	−0.14	0.10	−0.06	−1.42	0.157
Mother education level	−0.13	0.06	−0.10	−1.98	0.048 *
Father education level	0.10	0.07	0.08	1.50	0.133
Paternal acceptance	−0.10	0.02	−0.17	−4.05	0.001 ***
Paternal control	0.21	0.03	0.30	6.89	0.001 ***
Step 2—Parental Model					
Gender	−0.59	0.53	−0.05	−1.11	0.267
Age	−0.13	0.10	−0.05	−1.31	0.192
Mother education level	−0.14	0.06	−0.11	−2.20	0.028 *
Father education level	0.09	0.07	0.07	1.32	0.188
Parental acceptance	−0.13	0.03	−0.18	−4.02	0.001 ***
Parental control	0.23	0.04	0.29	6.54	0.001 ***

*Note.* Gender: 0 = female, 1 = male. Mother’s and father’s education levels represent completed years of education. To preserve space, results from three independent hierarchical regression analyses are presented in a single table. Sociodemographics in Step 1 are identical across all models. *p* < 0.05 *, *p* < 0.01 **, *p* < 0.001 ***.

**Table 6:** Results of Hierarchical Regression Analysis for Total Aggression Scores.

Variables	*B*	*SE*	β	*t*	*p*
Step 1					
Gender	4.45	1.82	0.11	2.45	0.015 *
Age	−1.01	0.34	−0.13	−2.94	0.003 **
Mother education level	0.08	0.22	0.02	0.39	0.698
Father education level	0.03	0.23	0.01	0.12	0.901
Step 2—Maternal Model					
Gender	4.54	1.75	0.11	2.60	0.010 *
Age	−0.97	0.33	−0.13	−2.93	0.004 **
Mother education level	0.08	0.21	0.02	0.37	0.712
Father education level	−0.05	0.22	−0.01	−0.23	0.819
Maternal acceptance	−0.32	0.10	−0.16	−3.32	0.001 ***
Maternal control	0.40	0.10	0.19	4.04	0.001 ***
Step 2—Paternal Model					
Gender	4.27	1.74	0.11	2.45	0.015 *
Age	−1.04	0.33	−0.14	−3.17	0.002 **
Mother education level	0.15	0.21	0.04	0.72	0.471
Father education level	0.00	0.22	0.00	0.02	0.983
Paternal acceptance	−0.17	0.08	−0.10	−2.18	0.029 *
Paternal control	0.59	0.10	0.26	5.92	0.001 ***
Step 2—Parental Model					
Gender	4.44	1.72	0.11	2.57	0.010 *
Age	−1.02	0.33	−0.13	−3.12	0.002 **
Mother education level	0.11	0.21	0.03	0.55	0.583
Father education level	−0.02	0.22	0.00	−0.09	0.926
Parental acceptance	−0.30	0.10	−0.13	−2.92	0.004 **
Parental control	0.64	0.11	0.25	5.63	0.001 ***

*Note.* Gender: 0 = female, 1 = male. Mother’s and father’s education levels represent completed years of education. To preserve space, results from three independent hierarchical regression analyses are presented in a single table. Sociodemographics in Step 1 are identical across all models. *p* < 0.05 *, *p* < 0.01 **, *p* < 0.001 ***.

## Data Availability

The original data presented in the study are available in Open Science Framework (osf.io/32czy).
